# *Aleurocanthus spiniferus* (Hemiptera: Aleyrodidae) in Some European Countries: Diffusion, Hosts, Molecular Characterization, and Natural Enemies

**DOI:** 10.3390/insects11010042

**Published:** 2020-01-07

**Authors:** Francesco Nugnes, Stefania Laudonia, Giovanni Jesu, Maurice Gerardus Maria Jansen, Umberto Bernardo, Francesco Porcelli

**Affiliations:** 1 National Research Council (CNR), Institute for Sustainable Plant Protection, 80055 Portici, Italy; umberto.bernardo@ipsp.cnr.it; 2 Department of Soil, Plant and Food Sciences, University of Bari Aldo Moro, 70126 Bari, Italy; francesco.porcelli@uniba.it; 3 Department of Agriculture, University of Naples Federico II, 80055 Portici, Italy; laudonia@unina.it (S.L.); giovanni.jesu@unina.it (G.J.); 4 Plant Protection Service, Section Entomology, 6700 HC Wageningen, The Netherlands; m.g.m.jansen@minlnv.nl

**Keywords:** CBC, *Citrus*, grape, stone and pome fruit tree pest

## Abstract

After the first record in 2008 in Southeast Italy, the alien invasive and quarantine pest *Aleurocanthus* *spiniferus* (orange spiny whitefly—OSW) has gradually spread throughout Europe, infesting several new host plants in addition to the known hosts. Molecular characterization of some Italian populations and a newly found Albanian population highlighted two different haplotypes invading Europe, belonging to one of the haplogroups previously recorded in China. A predator was recorded for the first time in several fields in Italy in association with OSW and other whitefly species. It was successively identified through a morpho-molecular characterization as a Nearctic member of the tribe Serangiini, the ladybird beetle, *Delphastus* *catalinae*. This predator represents a promising biocontrol agent to manage *A.* *spiniferus* outbreaks in Italy and other invaded countries.

## 1. Introduction

In Europe, more than 14,000 alien species have been recorded [1], half of which have become invasive. Their number is continuously rising [2] with a simultaneous increase in their diffusion rate [3]. Due to habitat fragmentation creating abundant and diverse niches, Italy is one of the most welcoming territories in Europe for foreign species. Many invasive insect species have been recently reported from Italy [4,5,6,7,8].

*Aleurocanthus spiniferus* (Quaintance) (Hemiptera: Aleyrodidae), the orange spiny whitefly (OSW), originating from China and South and Southeast Asia, is one of the serious pests infesting citrus [9]. Since its description [10], in the span of a century, OSW spread throughout Asia, Africa, Australia, and in the Pacific islands [11,12,13,14,15,16]. OSW was reported for the first time in the European and Mediterranean Plant Protection Organization (EPPO) areas after its first detection in the Lecce District (Apulia region, Southeast Italy) in 2008 [17]. Since then, OSW spread in the Apulia region, invading other municipalities neighboring Lecce [18] and, expanding northward, reached Brindisi and the districts of Bari and Taranto [15,19]. Although the spread of this pest was limited solely to the south-eastern area of Italy for about a decade, in June 2017 *A. spiniferus* was found in Salerno (Southwest Italy) [20]. Concurrently, OSW was also detected in the Balkan Peninsula including Croatia (2012), Montenegro (2013), and Greece (2016) [16,21,22].

OSW is considered one of the major threats to citrus production in Asia, Australasia, and the Nearctic zone [14,15,23]. The risk is mainly related to its high polyphagy as well as its self-spreading ability. OSW infests about 90 plant species belonging to 38 different plant families. In the area of the first European record, OSW was found on several hitherto unreported host-plants, among which some ornamentals were economically relevant: *Hedera helix* L., *Laurus nobilis* L., *Punica granatum* L., *Malus* spp., and *Prunus* spp. [15].

OSW infestations can weaken plants due to both direct and indirect damage ascribable to sap loss and the production of honeydew respectively. The remarkable amount of excreted honeydew encourages the growth of sooty mould, having negative effects on the photosynthetic process due to the copious soiling of the leaf surface [11,13].

Chemical control against OSW is not effective [24] and, in many cases, the frequent use of chemicals can adversely influence the natural enemy populations. The improper timing of treatments seems to be counter-productive, increasing the severity of infestation [25], probably due to side effects of pesticides on beneficial organisms.

The success of natural enemies against OSW in classical biological control (CBC) programs is widely recognized [11,26,27,28]. Several studies conducted in the native OSW territories highlighted the presence of a large group of natural enemies, including predators, parasitoids, and pathogens [29]. Predators recorded on *A. spiniferus* include species belonging to Diptera, Neuroptera, and a dozen ladybeetles [30]. However, most of the listed species have strongly polyphagous behavior [29] and OSW enemies also control the congeneric *Aleurocanthus woglumi* [15,30,31]. However, the complex of useful organisms that control OSW has been enriched because two other species native to the Palearctic region were recorded as preying on OSW in Italy: *Clitostetus arcuatus* (Rossi) (Coleoptera: Coccinellidae: Coccidulinae) [15], which is a specialist predator of whiteflies, and, sporadically, *Oenopia conglobata* (L.) (Porcelli, Pers. Comm.), which mainly preys on aphids (Hemiptera: Aphididae) and psyllids (Hemiptera: Psyllidae) [32,33].

Focusing on hymenopteran parasitoids, more than 10 wasp species were collected on OSW populations around the world [29,30,31]: most of them belong to the Aphelinidae family (*Ablerus connectans* Silvestri, *Encarsia smithi* (Silvestri), and *Eretmocerus* spp.), whereas only one Platygastridae (*Amitus hesperidum* Silvestri) was recorded. In the country of origin, some entomopathogenic fungi have been reported [29,34,35,36], but their role is still not well defined probably due to their poor specificity.

The recent OSW findings in several localities in Italy outline the relentless progress of its spread in the country [20]. Hence, we aimed to define biological and ethological aspects of OSW through: (1) providing an update of the distribution of OSW in the EPPO area, (2) revising the host plant list in the new areas of colonization, (3) evaluating the existence of genetic variability between populations from different areas and different host plants, and (4) finding and characterizing natural enemies in the newly infested areas with the object of evaluating the control of invasive populations of OSW.

## 2. Materials and Methods

### 2.1. Monitoring Activities

Since the first record in Southwest Italy (June 2017), monitoring activities were regularly conducted during the 2017–2019 to assess the presence and the spread of OSW in Campania [37]. Already known host plants were checked for the presence of all developmental stages in specialized and non-specialized orchards, private and urban gardens, ornamentals, and park areas. Similar inspections were completed of non-host plants close to infested plants, especially on wild plants or in abandoned fields, to evaluate the infestation of new plant species unrecorded as a suitable host. Unofficial monitoring was completed in places visited for other activities both in Italy and abroad.

Leaves infested by OSW were collected during the monitoring period, placed in sealed plastic bags in a refrigerated container, and carried to the laboratory. OSW samples were collected in eight localities from different host plants (Table 1). Specimens of different young developmental stages were removed with the help of a brush from the leaves, killed in absolute ethanol, and stored at −20 °C until analysis. Beetles, wasps, lacewings, and flies found on the *A. spiniferus* colonies were collected and placed on OSW-infested leaves in Petri dishes (25 ± 2 °C; 55% relative humidity, RH) to assess their role as natural enemies. Once we determined their ability or lack thereof against OSW, the inspected insects were collected or discarded, respectively. Collected specimens were treated as OSW specimens until analysis.

### 2.2. Morpho-Molecular Characterization

Samples were collected on all different recorded host species and on different plants in eight localities to evaluate the genetic diversity. For each locality and host species, a maximum of five specimens were used for the molecular analysis (Table 1).

*Aleurocanthus spiniferus* DNAs were extracted from each specimen using a non-destructive Chelex 100 (Bio-Rad, Richmond, CA, USA) and proteinase-K-based method as reported by Gebiola et al. [38].

After DNA extraction, OSW samples were rinsed in deionized water, slide-mounted as described by Cioffi [15], and identified following the relevant taxonomic descriptions [31,39,40,41,42,43].

The mitochondrial gene *cytochrome c oxidase subunit I* (*COI*) was amplified using the primer pair AsFmik and AsR4mik [44] with the PCR profile reported by Uesugi [9].

For samples of *Delphastus catalinae* Horn 1895, due to the absorption of the solution during the DNA extraction process, the method described by Gebiola et al. [38] was slightly modified as follows. We used 6 μL of 20 mg/mL proteinase-K and 100 μL 5% Chelex 100 suspension to obtain DNA from single wild beetles collected in some fields in Campania and Sicily, feeding on OSW and *Aleurothrixus floccosus (Mask.)* colonies, respectively (Table 2). Samples supplied from two biofactories and commercialized as *D. catalinae* and *D. pusillus* (LeConte, 1852) (Table 2) were included in this study. Some samples were previously observed through the use of a Cryo-SEM (Hitachi (Tokyo, Japan) TM 3000 series). This methodology does not require critical point drying or metal coating, and the same observed sample can be later submitted to DNA extraction or/and conventional slide mounting.

Once rinsed in deionized water, some beetle specimens were dissected and mounted on slides using balsam-phenol as a permanent medium; others were mounted on cards.

Extracted DNA was employed to amplify a portion of the mitochondrial gene *COI* using the forward primer C1-J-2183 with the reverse primer TL2-N-3014 [45] following the thermocycler conditions described in Gebiola et al. [38].

PCR products were checked on a 1.2% agarose gel stained with GelRED^®^ (Biotium, Fremont, CA, USA) and directly sequenced. Sequences were assembled and edited by eye with Bioedit 7.2.5 [46], and were virtually translated into the corresponding amino acid chain to detect frame-shift mutations and stop codons, using EMBOSS Transeq (http://www.ebi.ac.uk/Tools/st/emboss_transeq/ (accessed 23 October 2019)). Edited sequences were checked against the GenBank database and were submitted to the GenBank database under accession numbers reported in Table 1 and Table 2.

*Aleurocanthus spiniferus COI* genetic distances and standard errors (SE) were calculated with MEGA 6 software [47] as uncorrected *p*-distance considering homologous sequences of OSW available in GenBank (accessed 23 October 2019).

The relationships between *A. spiniferus* specimens were also investigated using Statistical Parsimony in TCS 1.21 [48] on the *COI* dataset.

OSW phylogeny was reconstructed using maximum likelihood (ML) in RAxML 7.0.4 [49]. A GRT+G+I nucleotide substitution model was used, as selected by jModeltest [50]. ML branch support was based on 1000 rapid bootstrap pseudoreplicates, and clades were considered supported when bootstrap values were >70%. Homologous sequences available in GenBank were included in the alignment and the tree was rooted including the congeneric *A. camelliae* (Kanmiya & Kasai) sequences.

## 3. Results

### 3.1. Monitoring Activities

The survey results indicated that in the new areas of colonization, *A. spiniferus* was recorded on the main elective hosts *Citrus* spp. and on the already known host plants, *Ceratonia siliqua* L., *Eriobotrya japonica* (Thunb.) Lindl., *Hedera helix* L., *Morus alba* L., *Prunus armeniaca* L., *Punica granatum* L., *Rosa* spp., and *Vitis vinifera* L. OSW was collected from several additional host plants belonging to several botanical families that represent new associations (Table 3).

In October and December 2017, findings in two different sites in Salerno of some small coleopteran belonging to the Coccinellidae revealed some small ladybeetles preying on *A. spiniferus* populations infesting leaves of *C. limon* and *R. banksiae*. Subsequent surveys in the same areas resulted in the collection of all ladybird developmental stages, from eggs to adults.

### 3.2. OSW Characterization

Mitochondrial *COI* sequencing revealed the presence of two haplotypes in the sampled *A. spiniferus* (Table 1). BLAST search revealed that both obtained haplotypes belong to mitochondrial haplogroup 2 [9] corresponding to the haplotypes H1 and H2 recently found in Greece, and Greece, Italy, and Montenegro, respectively [16].

Haplotype H1 was obtained from samples collected in Bari, Rome, Mattinata (Foggia), San Gennaro Vesuviano (Naples) and in four out five samples from Pescara. Haplotype H2 was detected in other samples from Bari and Pescara, and in all analyzed samples from Salerno, Portici (Naples), and Buthrotum (AL). The percentages of detection of haplotypes were 57% for H1 and 43% for H2. MEGA analyses highlighted that the mean intra-group distances existing between the haplotypes belonging to the haplogroup 2 was 0.31% (±0.002% SE), and 0.8% (±0.003%) in haplogroup 1. Focusing on haplogroup 2, the distance between H1 and H2 sequences recovered in this study was 0.6% (±0.003%), corresponding to four variable and parsimony-informative sites. The inter-group distance between haplogroups 1 and 2 was 12.1% (±0.017%).

**Figure 1:**
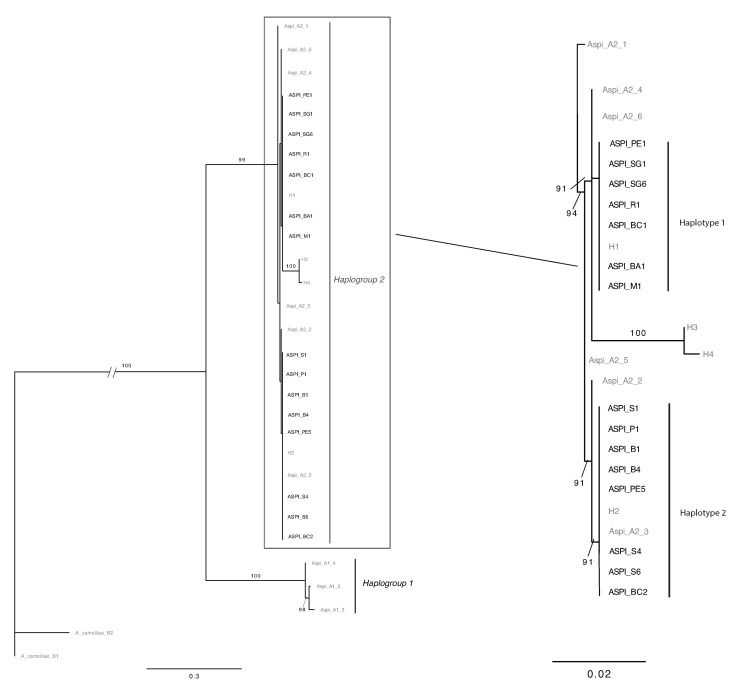
Maximum likelihood trees based on *COI* sequences of *A. spiniferus*. Bootstrap values >70% are shown above the branches. Complete tree (**left**) and zoom on phylogenetical relationships in Haplogroup 2 (**right**). Sequences from [9] and [16] are in grey; sequences obtained in this work are in black.

Phylogenetic reconstruction resulted in an ML tree (Figure 1) where the two haplogroups were identified in two highly supported clades. The statistical parsimony with TCS yielded two separate networks, corresponding to the haplogroup 1 and haplogroup 2. The connection limit necessary to obtain a single network was 77 steps.

### 3.3. Natural Enemies

One single predator was reared from *A. spiniferus* colonies and a preliminary morphological identification identified the collected ladybeetles as *D. catalinae*. The sequenced region of the mitochondrial *COI* gene of the Italian sample and those provided by biofactories (Table 3) were identical to each other. The BLAST analysis of this *COI* sequence showed a 100% similarity to the *D. catalinae* sequence presents in GenBank (Accession number MF152800).

## 4. Discussion

*Aleurocanthus spiniferus* was found on several host plant species in the new areas of colonization confirming previous surveys [15]; in addition, 11 new hosts were found. These findings highlight the already known polyphagy of OSW, which could accelerate its spread in territories where the main hosts are absent, permitting its quick spread in Italy and Albania. This is the first record of OSW in Albania.

**Figure 2:**
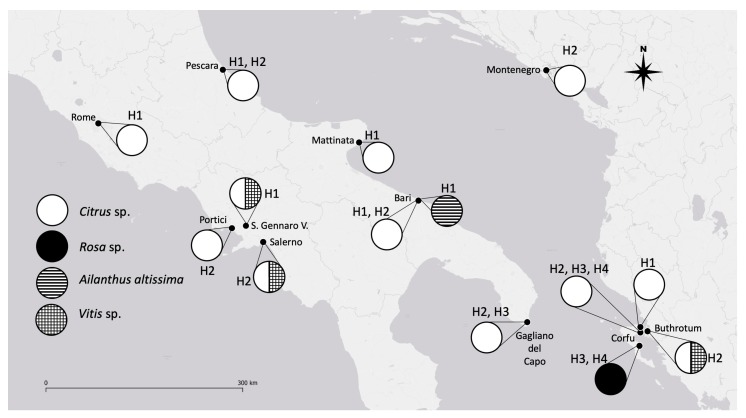
*A. spiniferus* distribution, mitochondrial haplotypes, and respective host plants in EPPO zone. EPPO, European and Mediterranean Plant Protection Organization areas.

In the present study, only two haplotypes (H1 and H2) were found, different from what was recently found in Greece, where four different haplotypes were recorded [16]. Therefore, some conclusions are possible (Figure 2):

(1) The H4 haplotype seems to have a reduced diffusion (present only in Greece) [16] because it was not found in any other of the collection areas (Italy and Albania).
(2) The H3 haplotype was previously found in Apulia [16] in the area of the first interception of OSW but it was not found in the present study. This could be due to two possible causes: a poor diffusion of this haplotype (20% of the survey in Italy) [16] and a predominance of the other haplotypes.
(3) The H1 haplotype was recovered in Apulia (both in Bari and in Mattinata) during our sampling but it was not found in the study performed by Kapantaidaki [16]. The different compositions of haplotypes in the samples collected by different authors could be linked both to different sampling methodologies and/or to a patch-like distribution of the different haplotypes.

The reduced genetic variability of OSW specimens collected in the EPPO area (four haplotypes), in the present and previous work [16], compared to those found in the native country (12 haplotypes) [9] may be due to the founder effect that affects invasive species. This is a common pattern for invasive species whose population is established by a few specimens [4,51,52,53]. Interestingly, three out of four haplotypes (H1, H3, and H4) are not found in the country of origin [9,16]. Among them, H1 is the most widespread in European invaded countries. This scenario is similar to the spread of the eucalyptus gall wasp, *Leptocybe invasa* Fisher and La Salle, in which the main globally spreading haplogroup was never found in the native territories [54]. H4 was found only in Greece, where several different haplotypes have also been found. This finding suggests that the population that invaded Greece may act as a bridgehead for the subsequent introductions to the other countries; however, based on the invasive history of OSW in Europe, this scenario is not temporally plausible [16]. Therefore, the most well-founded hypothesis is that multiple introductions of this species have occurred in the EPPO area. However, for a definitive confirmation of this hypothesis, a wider sampling is necessary.

Our results definitively exclude the possibility that a specificity of a haplotype exists for a host plant because both haplotypes were collected on several different host plants (Table 1 and Figure 2).

Differently, during the growing season, *A. altissima* hosted only specimens with mt-H1, whereas *Vitis* sp. hosted solely a population with the mt-H2 haplotype. Both *Vitis* sp. and *A. altissima* are deciduous plants and, therefore, during the winter, they cannot host OSW populations, so these species are re-colonized only in the next spring. However, their role (as that of other deciduous trees hosting OSW) is probably crucial in the increase in OSW populations. In spring–summer, such plants could produce a “flywheel effect” in increasing the adult population overwintering on evergreen plants, increasing the chances of survival of winter, thus enabling severe infestations during the following spring.

The finding of OSW on *A. altissima* requires more detailed studies. The only whitefly reported on the tree of heaven is *Dialeurodes citri* (Ashmead), whereas congeneric species *A. excelsa* (Roxb.) seems to be a well-known host for at least four different genera of whiteflies [30,55,56] and OSW is not included.

Studies of genetic distances, supported by phylogenetic analysis and statistical parsimony, indicated a high genetic distance between the two haplogroups (12.1%). These results are consistent with a previous study [9], providing strong indication that the two haplogroups should be reevaluated through an integrative approach because they could result in different species. An integrative approach, considering other molecular markers, biological characteristics, and morphometric analyses, often allows the delimitation and description of different species previously considered single species [53,57,58,59]. The genetic diversity could have important implications in the management of the pest because natural enemies could have different specificity toward distinct cryptic species [60]; for example, the different biology of two pests could affect the approach necessary for their management [61].

During our survey, only a single predator (*D. catalinae*) was recorded and our samplings highlighted the presence of different developmental stages of *D. catalinae* feeding on *A. spiniferus* populations.

*Delphastus catalinae* is a polyphagous species that has been previously recorded on several prey species: *A. floccosus*, *Pealius kelloggi* (Bemis), *Dialeurodes citri*, *D. citrifolii* (Morgan), *Bemisia tabaci* (Genn.), *Aleurodicus dispersus* Russel, *Trialeurodes vaporariorum* (Westwood), and *A. woglumi* [62,63].

The genus *Delphastus* Casey, belonging to the tribe Serangiini, is native to the Nearctic region and does not include any species native to Europe [62,63]. All the members of the tribe are obligate whitefly predators [62,64,65] and, due to their use as biological control agents, are mass-produced in the USA [66]. Gordon [62] defined *D. catalinae* distribution as “an artificial distribution that includes South, Central, and North America, as well as the Canary Islands and Hawaii […] probably results from commercial trade”. The sequences of all the examined specimens, even if of different origins, were all identical, which could be evidence that the population found in Italy was derived from field releases and therefore from a biofactory. Booth and Polaszek [63] based their comments about the species on additional laboratory material from Israel and the Netherlands (cultures from Israel) and the U.K. (cultures from Canada). In addition, a similar species, *D. pusillus*, was released in several augmentative biological program attempts [67,68]. However, because our results demonstrated that the species considered *D. pusillus* reared in commercial insectaries was instead *D. catalinae*, only the latter was probably to date used in biological control programs [63,69]. Correct identification of species reared and employed in the biological control program is crucial [70]. However, the identification (especially of small and live specimens) can lead to rearing and introducing incorrectly identified species if recently revised identification protocols are not used or the identification is not confirmed by a taxonomic specialist. An erroneous release could also occur when biofactories either rear congeneric species or introduce wild specimens to avoid the negative effect of prolonged inbreeding [58]. Several problems related to the small size of this species, some clearing and mounting artefacts, and to the high release of *D. pusillus* in several augmentative biological programs around the world, necessitate a re-description of the species.

**Figure 3:**
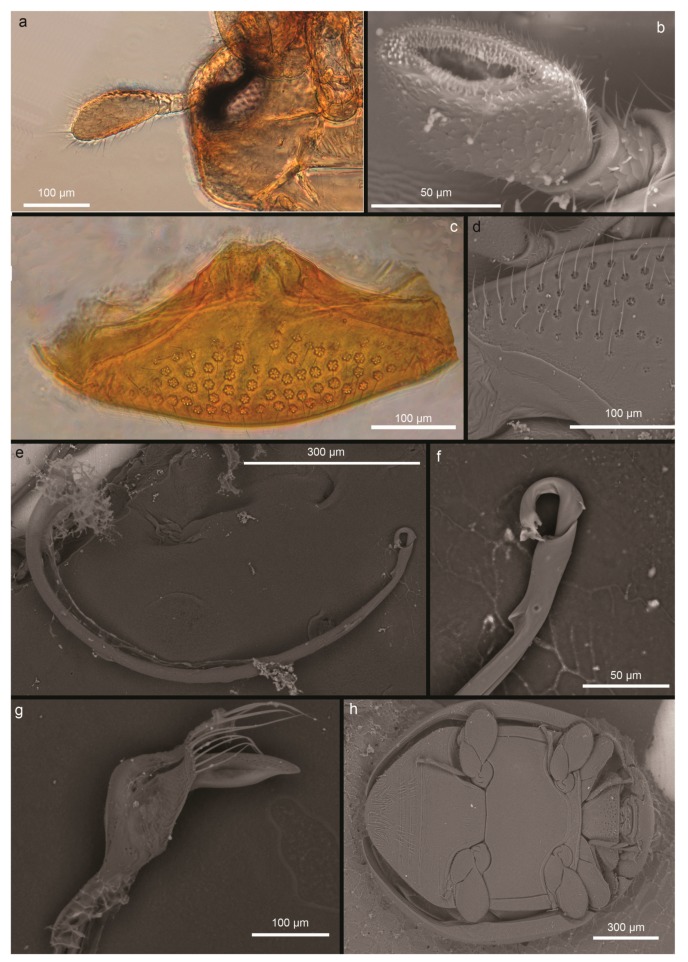
*Delphastus catalinae* morphological characters. (**a**) antenna, (**b**) apical segment of maxillary palp, (**c**) prosternum, (**d**) particular of the setae encircled by loculi, (**e**) sipho, (**f**) tip of the sipho, (**g**) median lobe of parameres, and (**h**) body (ventral side).

As well as other members of the tribe Serangiini, the genus has a small body that is ovoid and strongly convex at the dorsum [70]. In particular, *D. catalinae* presents a one-segmented antennal club, 2.2 times longer than wide (Figure 3a); maxillary palp with apical segment conspicuous, two times longer than wide, ovoid, with the inner face truncated in oblique where sensilla are placed (Figure 3b); prosternum shows dense setose punctures (*n* = 10) consisting of seta, each encircled by not less than five loculi (Figure 3c,d). Female length is 1.38 ± 0.17 mm (*n* = 10); males are 1.22 ± 0.1 mm (*n* = 10) in length. Male genitalia are asymmetrical, phallobase with unpaired apodeme, and sipho arcuate with spathiform siphonal proximal capsule (Figure 3e,f); the parameres are very short but recognizable and with long setae, which reach the apex of the median lobe (Figure 3g); legs with expanded femurs, middle and hind tibiae are arcuate with bristles on outer margin, and tarsus three-segmented (Figure 3h).

Some preliminary tests conducted on leaves of *C. limon* infested with OSW in the laboratory confirmed *D. catalinae* preys on OSW. Studies are underway to examine the performance of the predator on crops in the field, its phenology, and the preferences of the hosts and plants in the new colonized environments. The activity of natural enemies will probably be exploited by employing proper conservation and augmentation techniques.

## 5. Conclusions

*Aleurocanthus spiniferus* is still spreading in the Mediterranean Basin, invading new areas and infesting new host plants. This invasive species has the potential to strongly affect the production and development of some species common in the Mediterranean orchards and gardens.

After the first record in 2008 in Southeast Italy, the alien invasive and quarantine pest *A. spiniferus* (Quaintance) (Hemiptera: Aleyrodidae) has gradually spread throughout Europe, infesting several new host plants in addition to the known hosts. Molecular characterization of some Italian populations and a newly found Albanian population highlighted only two different haplotypes invading Europe belonging to one of the haplogroups previously recorded in China. Through morpho-molecular characterization, the ladybird beetle *D. catalinae*, a Nearctic member of the tribe Serangiini, was recorded for the first time in fields in Italy in association with OSW and other whitefly populations. *D. catalinae* shows potential as a biocontrol agent to manage *A. spiniferus* outbreaks either in Italy or in other invaded countries. The finding of *D. catalinae* on several host plants feeding on the OSW population indicates the possibility for an eco-compatible solution to control this threatening phytophagous insect, but evaluations on its field effectiveness are still in progress.

## Figures and Tables

**Table 1:** Information about the specimen involved in this study and respective haplotyping results and sequences accession numbers. FG, Foggia; NA, Naples.

Specimen Code	Location	Coordinates	Host Plant	Date of Record	Haplotype	Accession Number
ASPI PE1	Pescara	42°27′ N 14°12′ E	*Citrus* sp.	16 September 2019	H1	MN662884
ASPI PE2					H1	MN662885
ASPI PE3					H1	MN662886
ASPI PE4					H1	MN662887
ASPI PE5					H2	MN662925
ASPI R1	Rome	41°54′ N 12°29′ E	*Citrus* sp.	7 March 2019	H1	MN662888
ASPI R2					H1	MN662889
ASPI M1	Mattinata (FG)	41°42′ N 16°04′ E	*Citrus* sp.	16 August 2018	H1	MN662917
ASPI M2					H1	MN662918
ASPI M3					H1	MN662919
ASPI BA1	Bari	41°06′ N 16°53′ E	*Ailanthus altissima*	7 August 2017	H1	MN662912
ASPI BA2					H1	MN662913
ASPI BA3					H1	MN662914
ASPI BA5					H1	MN662915
ASPI BC1		41°06′ N 16°52′ E	*Citrus* sp.		H1	MN662916
ASPI BC2					H2	MN662890
ASPI BC3					H2	MN662891
ASPI BC4					H2	MN662892
ASPI SG1	San Gennaro Vesuviano (NA)	40°51′ N 14°31′ E	*Citrus nobilis*	13 September 2019	H1	MN662893
ASPI SG2					H1	MN662894
ASPI SG3					H1	MN662895
ASPI SG4					H1	MN662896
ASPI SG5					H1	MN662897
ASPI SG6			*Vitis* sp.		H1	MN662898
ASPI SG7					H1	MN662899
ASPI SG8					H1	MN662900
ASPI SG9					H1	MN662901
ASPI SG10					H1	MN662902
ASPI P1	Portici (NA)	40°49′ N 14°19′ E	*Citrus limon*	14 February 2019	H2	MN662920
ASPI P2					H2	MN662921
ASPI S1	Salerno	40°40′ N 14°45′ E	*Citrus sinensis*	16 June 2018	H2	MN662922
ASPI S2					H2	MN662903
ASPI S3					H2	MN662904
ASPI S4			*Citrus reticulata*	16 May 2019	H2	MN662905
ASPI S5					H2	MN662906
ASPI S6			*Vitis* sp.		H2	MN662907
ASPI S7					H2	MN662908
ASPI B1	Buthrotum (Albania)	39°44′ N 20°01′ E	*Citrus* sp.	20 July 2018	H2	MN662923
ASPI B2					H2	MN662924
ASPI B3					H2	MN662909
ASPI B4			*Vitis* sp.	21 July 2018	H2	MN662910
ASPI B5					H2	MN662911

**Table 2:** Beetle specimens used in this study.

Specimen Code	Preliminary Identification	Origin or Commercial Product	Date of Record	Host-Plant/Host	Molecular Identification	Morphological Re-Examination	Accession Number
DC1	*Delphastus catalinae*	Salerno ^a^	21 October 2017	*Citrus limon*/*Aleurocanthus spiniferus*	*D. catalinae*	*D. catalinae*	MN662936
DC2							MN662937
DC3							MN662938
DC4							MN662939
DC-C1	*D. catalinae*	Delphibug ^b^	22 August 2018				MN662940
DC-C2							MN662941
DP1	*D. pusillus*	Delphastus-System ^c^	12 August 2018				MN662942
DP2							MN662943
DP NO1	*D. catalinae*	Noto (Sicily) ^a^	27 August 2018	*C. limon/Aleurothrixus floccosus*			MN662944

^a^ field sampling; ^b^ provided by Koppert; ^c^ provided by Biobest.

**Table 3:** Additional host-plant species found infested by *A. spiniferus* in the present study.

Host Plant Family	Host Plant Species
Simaroubaceae	*Ailanthus altissima* (Mill.) Swingle
Ericaceae	*Arbutus unedo* L.
Rutaceae	*Citrus medica* L.
	*Citrus reticulata* Blanco
Ranunculaceae	*Clematis vitalba* L.
Anacardiaceae	*Pistacia vera* L.
Rosaceae	*Prunus avium* (L.)
	*P. cerasus* L.
	*P. domestica* L.
	*Rosa banksiae* Aiton
	*R.* × *damascena* Herrm.
